# Potentiometric NO_2_ Sensors Based on Thin Stabilized Zirconia Electrolytes and Asymmetric (La_0.8_Sr_0.2_)_0.95_MnO_3_ Electrodes

**DOI:** 10.3390/s150717558

**Published:** 2015-07-20

**Authors:** Jie Zou, Yangong Zheng, Junliang Li, Zhongliang Zhan, Jiawen Jian

**Affiliations:** 1 Gas Sensors & Sensing Technology Laboratory, College of Information Science and Engineering, Ningbo University, Ningbo 315211, China; E-Mails: zoujievivid@163.com (J.Z.); zhengyangong@nbu.edu.cn (Y.Z.); 2 CAS Key Laboratory of Materials for Energy Conversion, Shanghai Institute of Ceramics, Chinese Academy of Sciences (SICCAS), Shanghai 200050, China; E-Mail: ljl2005@mail.sic.ac.cn

**Keywords:** NO_2_, sensors, lanthanum strontium manganite, yttria-stabilized zirconia, gas-phase reaction

## Abstract

Here we report on a new architecture for potentiometric NO_2_ sensors that features thin 8YSZ electrolytes sandwiched between two porous (La_0.8_Sr_0.2_)_0.95_MnO_3_ (LSM95) layers—one thick and the other thin—fabricated by the tape casting and co-firing techniques. Measurements of their sensing characteristics show that reducing the porosity of the supporting LSM95 reference electrodes can increase the response voltages. In the meanwhile, thin LSM95 layers perform better than Pt as the sensing electrode since the former can provide higher response voltages and better linear relationship between the sensitivities and the NO_2_ concentrations over 40–1000 ppm. The best linear coefficient can be as high as 0.99 with a sensitivity value of 52 mV/decade as obtained at 500 °C. Analysis of the sensing mechanism suggests that the gas phase reactions within the porous LSM95 layers are critically important in determining the response voltages.

## 1. Introduction

In lean-burn diesel engines the excess of air can increase the fuel efficiency [1], but may produce NO_x_ emissions that are supposed to be correlated with detrimental environmental observations such as photochemical smog, acid rain and respiratory syndrome [2,3]. NO_x_ sensors are typically installed in the selective catalytic reduction (SCR) system to minimize NO_x_ concentrations in the exhaust gases from the diesel and lean-burn Otto engines [4]. Recently, different types of NO_x_ sensors based on solid-state electrolytes have been reported, which commonly use Pt as the reference-electrodes (REs) and oxides as the sensing-electrodes (SEs) [5,6,7,8,9]. Among them, YSZ-based potentiometric NO_x_ sensors are especially attractive due to their desirable long-term stability and excellent sensing properties toward O_2_, NO_x_, hydrocarbons (HCs) and CO. However, these YSZ-based potentiometric sensors always exhibit lower potential difference (Δ*V*) in the planar configuration when compared to their tubular counterparts under the same conditions, resulting from the inevitable response of Pt-RE toward target gases [6,10]. To address these issues, Pt-loaded zeolite Y (PtY) is used as RE and micron-scale Pt as SE due to the high catalysis response toward NO_2_. The gas-phase decomposition of NO_2_ in the RE plays a key role on the sensing performance of the sensors [11,12,13]. However, the high costs and complicated fabrication procedures of Pt electrodes and PtY filters have prevented their practical applications. Therefore, it is necessary to search for inexpensive and efficient alternatives that can be easily fabricated as REs and SEs.

La_0.8_Sr_0.2_MnO_3_ (LSM) has been widely studied as a catalyst to decompose NO_x_ [14,15,16,17,18,19]. Additionally, LSM has also been considered as an ideal cathode material, which can be co-fired with thin YSZ electrolytes in solid oxide fuel cells (SOFCs) [20,21,22]. In comparison with the stoichiometric oxide, A-site deficient LSM exhibits reduced sintering temperatures while maintaining similar electrical and catalytic properties, suggesting that its detrimental interaction with 8YSZ electrolytes could be minimized by co-firing at lower temperatures [23,24,25]. Based on these observations, it is reasonable to replace the asymmetric Pt-SE/RE in YSZ-based potentiometric NO_2_ sensors with inexpensive A-site deficient LSM.

Recently, we have fabricated planar YSZ-electrolyte NO_2_ sensors with thick and thin (La_0.8_Sr_0.2_)_0.95_MnO_3_ (LSM95) layers as REs and SEs, respectively. In the meanwhile, the thick LSM95 layers were used as the device substrates. In this report, the sensing performances of new potentiometric NO_2_ sensors with different porosity REs and different SEs were compared, respectively. The preliminary results showed that the very thick LSM95 layer plays an important role in determining the sensing performance. The very different thicknesses and diffusion pathways between the two LSM95 layers allowed for good NO_2_ sensing properties at high temperatures.

## 2. Experimental Procedure

### 2.1. Synthesis of Electrode Powders

A-site deficient LSM95 powders were synthesized using the conventional solid-state reaction method. Analytical grades of La_2_O_3_ (Ruier, Guangzhou, China), SrCO_3_ (Sinopharm Chemical Reagent, Shanghai, China) and MnCO_3_ (Sinopharm Chemical Reagent) were used as the starting materials. These raw materials were mixed in the stoichiometric ratio for 2 h in a planetary mill using triethanolamine (TEA), ethanol and zirconia balls as the dispersant, solvent and milling medium, respectively. This mixture was dried at 80 °C and then calcined at 1050 °C for 4 h.

### 2.2. Fabrication of Sensors

**Figure 1:**
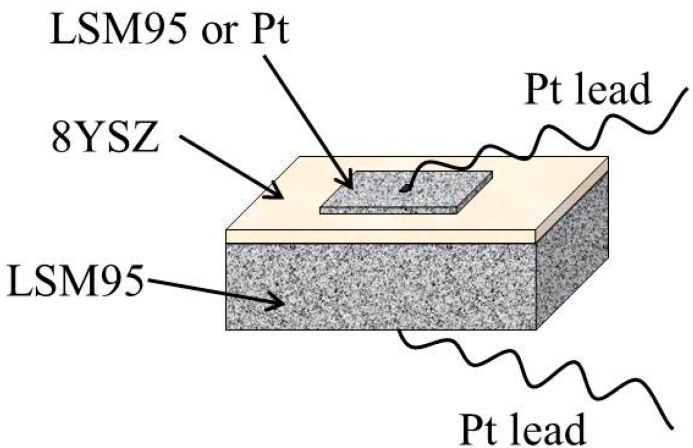
Schematic illustration of the NO_2_ sensors.

Commercially available 8YSZ powders (Tosoh, Yamaguchi, Japan) were used as the electrolytes and self-synthesized LSM95 powders were used to prepare the RE substrates using the tape casting technique with carbon (Cancarb, Medicine Hat, AB, Canada) as the pore former. Screen printing was used to prepare SE from LSM95 ink and commercial Pt ink (Sino-platinum, Kunming, China). The tape casting slurries were prepared by a two-step ball milling procedure. In the first step, the targeted ceramic powders were homogeneously dispersed in a planetary mill for 1 h using zirconia balls, acrylic and xylene/butyl acetate as the milling medium, dispersant and solvents, respectively. Then, polyvinylbutyral (PVB) was added as the binder and polyethylene glycol (PEG) as the plasticizer, followed by another planetary milling for 2 h. The content of carbon in the LSM95 slurries were 15, 30, 45 and 60 wt % relative to the ceramic powders, respectively. After de-airing for 10 min under vacuum, the LSM95 and 8YSZ slurries were cast onto Mylar substrates with a doctor blade height of 300 or 75 μm. After drying, the green LSM95 and 8YSZ tapes were about 125 and 30 μm, respectively. In order to fabricate the LSM95 supported sensors, one sheet of 8YSZ green tape was laminated with the six similar sheets of LSM95 green tape. The LSM95 powders were mixed with α-terpineol to prepare print ink. The LSM95 ink was printed onto the top of green 8YSZ tapes. The sensors were denoted as S-15LSM95, S-30LSM95, S-45LSM95 and S-60LSM95. The Pt ink was printed onto the top of green 8YSZ tapes using the thick LSM95 layer with 15 wt % carbon as RE. The resultant sensors were denoted as S-15Pt. These green sensors were co-fired in air at 1225 °C for 4 h with a ramp rate of 3 °C/min. The schematic of NO_2_ sensors is showed in Figure 1. As the electrode leads, Pt wires were bonded to the electrode surfaces using a small amount of Pt ink. The particle size and pore size distributions were measured by laser particle analyzer (Mastersizer 2000, Malvern, Worcestershire, UK) and mercury intrusion porosimeter (Micromeritics AutoPore IV 9500, Norcross, GA, USA). The phase composition and microstructures of sensors were examined by X-ray diffraction with Cu-Kα radiation (XRD, D8 advance, Bruker, Billerica, MA, USA) and scanning electron microscopy (FE-SEM, SU-70, Hitachi, Tokyo, Japan).

### 2.3. Evaluation of the Sensing Performances

To evaluate the sensing characteristics of the sensors at 500–600 °C, gas sensing experiments were carried out in a conventional gas-flow apparatus with a single chamber. Both thin LSM95 layers and thick LSM95 layers were simultaneously exposed to the base gas (5 vol % O_2_ + N_2_ balance) or the sample gas containing each of various gases (NO_2_, NO, NH_3_, CH_4_, C_3_H_6_, H_2_, 400 ppm each in the base gas or NO_2_ over 40–1000 ppm in the base gas).

**Figure 2:**
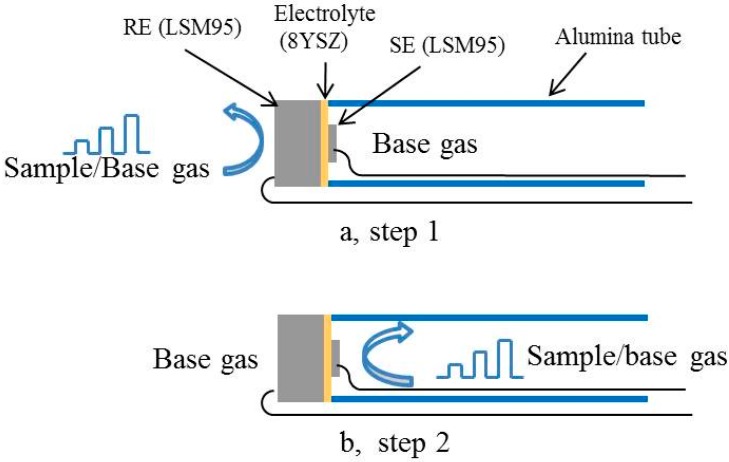
Schematic illustration of NO_2_ sensors measured in the two-chamber mode.

In order to individually evaluate the sensing performances of thick and thin LSM95 layers toward NO_2_, S-15LSM95 was also measured in a two-chamber mode. The measurement was first performed with thin LSM95 layers exposed to the base gas while the thick LSM95 layers switched between the sample gas and the base gas, as shown in Figure 2a. In the second step, the environments were reversed with the thick LSM95 layer exposed to the base gas and the thin LSM95 layer to either the sample or base gas (Figure 2b). Note that the lag time between the sample gas and the base gas was always held for 10 min. The total gas flow rate was maintained at 200 cm^3^/min. The voltage responses were recorded by using a multifunction data acquisition card (HP 34970A, Santa Clara, CA, USA).

## 3. Results and Discussion

### 3.1. Properties of LSM95 and Carbon Powders

**Figure 3:**
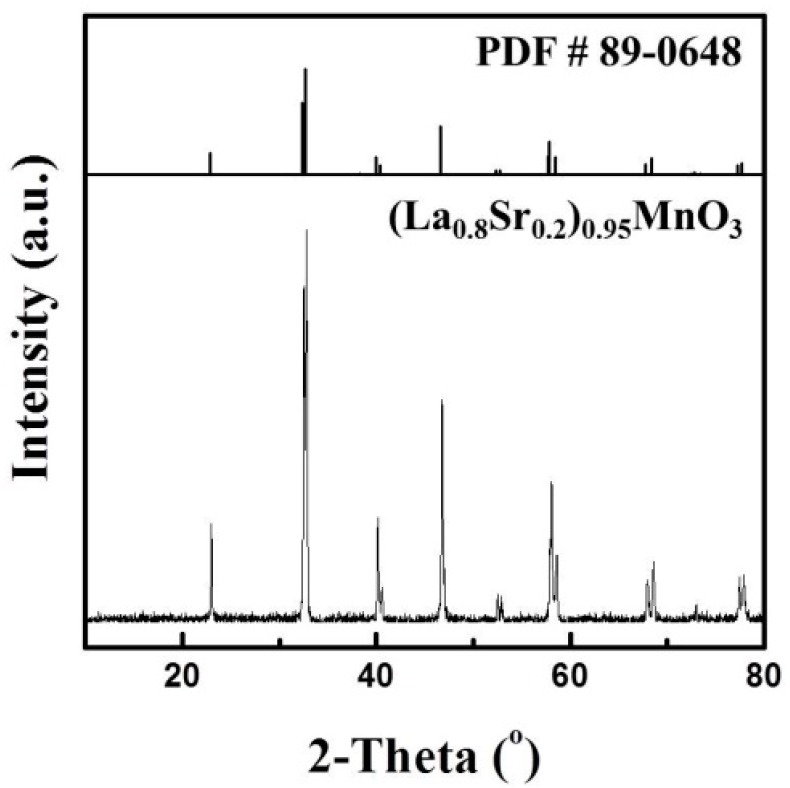
XRD patterns of LSM95 powders.

**Figure 4:**
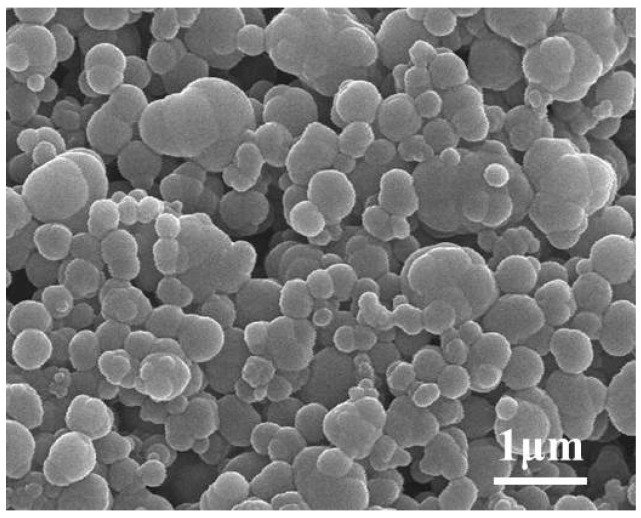
SEM micrographs of carbon.

**Figure 5:**
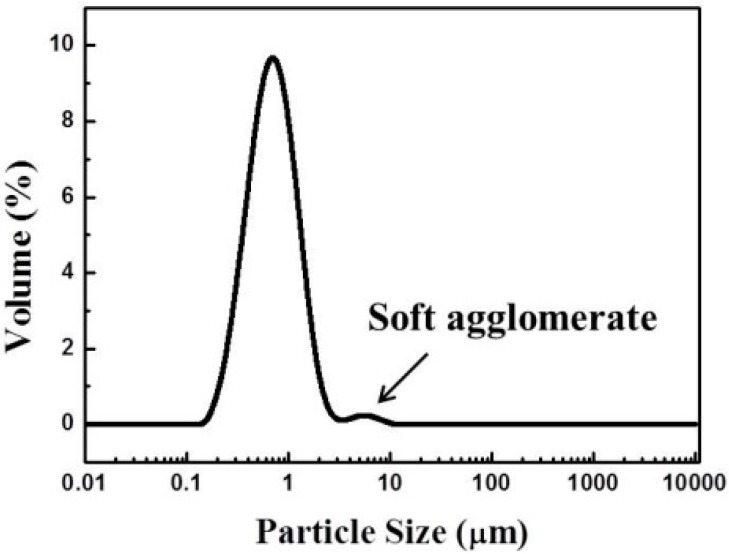
Particle size distribution of carbon.

Figure 3 shows the X-ray diffraction (XRD) pattern of the as-prepared LSM95 powders, which can be well indexed according to the standard XRD pattern, JCPDS PDF NO. 89-0648. The calculated crystallite size using the Scherer formula is about 83.7 nm. Chemical compatibility between LSM95 and 8YSZ was confirmed by X-ray diffraction patterns of their composites calcined at 1150–1350 °C, where no additional peaks from impurities were observed [26]. Figure 4 shows the SEM micrograph of carbon which was used as pore former in the thick LSM95 layers and the particles are spherical granules. The particle sizes of carbon were also examined with results are showed in Figure 5. Two peaks can be observed with a large one at around 600 nm and the small one is at 4000 nm, where the former resulted from primary particles and the latter was due to undispersed soft agglomerates. Figure 5 shows that the size of spherical granules largely distributed from 150 to 1500 nm and with the mean diameter (D50) at 631 nm.

### 3.2. Microstructure of Sensors

**Figure 6:**
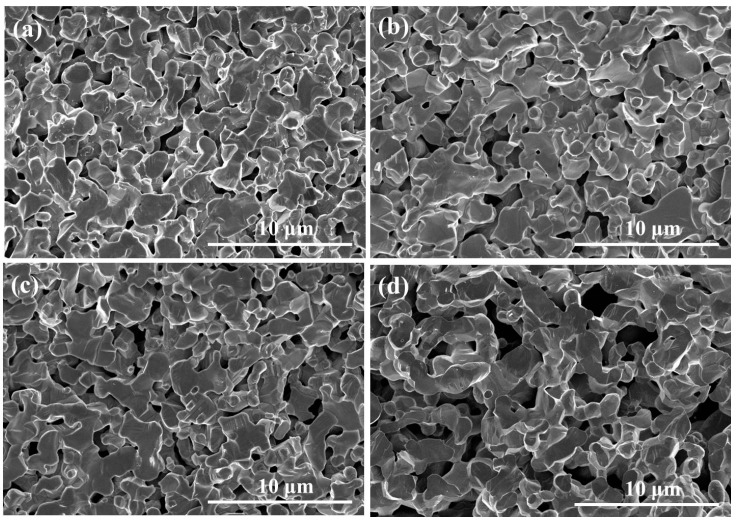
SEM micrographs of thick LSM95 layers, (**a**) 15 wt % carbon; (**b**) 30 wt % carbon; (**c**) 45 wt % carbon; (**d**) 60 wt % carbon.

**Figure 7:**
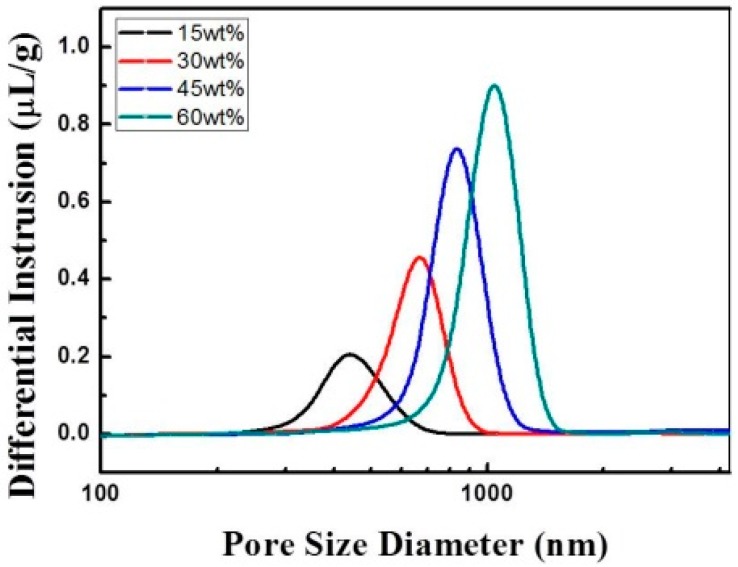
Pore size distribution of thick LSM95 layers.

Figure 6a–d show the microstructure of thick LSM95 layers with different amounts of pore former (15, 30, 45 and 60 wt %). Both the pore size and porosity increased gradually with increasing of pore formers, as confirmed by the mercury porosimetry measurements shown in Figure 7. In particular, the mean pore sizes are 430, 680, 820 and 1000 nm and the porosities are 20.9%, 41.9%, 50.1% and 65.2% for LSM95 substrates with 15, 30, 45 and 60 wt % carbon, respectively.

**Figure 8:**
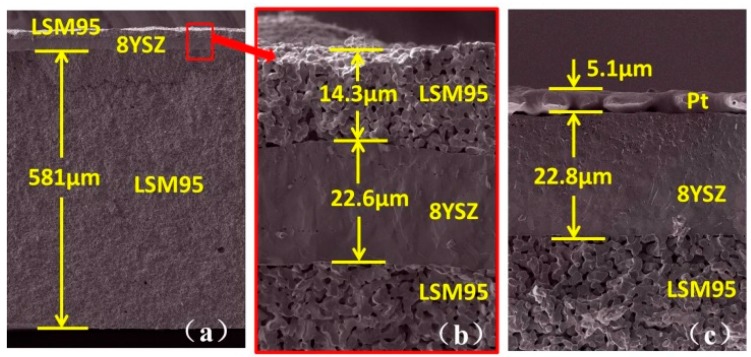
SEM micrographs of sensors, (**a**) Low magnification view of fractured S-LSM95; (**b**) High magnification view of S-LSM95; and (**c**) High magnification view of S-Pt.

Figure 8a shows a representative cross-sectional SEM micrograph of the new S-15LSM95 sensors with thin LSM95 layers as SEs, where the thick LSM95 layers as REs are ≈580 μm thick. By the way, all sensors have similar thickness of REs. Higher-magnification view (Figure 8b) shows that both LSM95 layers display similar porous microstructures with high enough porosity to facilitate the gas transport. Note that the 8YSZ electrolytes are fully dense after firing at 1225 °C, which is 100–200 °C lower than the commonly required sintering temperature. Such observations can be explained by the large shrinkage (>23%) of the thick LSM95 layers at lower sintering temperatures, which is conducive to the densification of thin YSZ electrolytes [23]. Figure 8b also shows that the thickness is 14 μm for thin LSM95 layers and 23 μm for YSZ electrolytes. For the NO_2_ sensors S-Pt with thin Pt layers as SEs, the microstructure of the supporting LSM95 substrates (Figure 8c) are largely the same as observed for S-LSM95. The Pt layers are ≈5 μm thick and contained relatively large pores due to the excessive sintering during the co-firing process.

### 3.3. Influence of Porosity on Sensing Performance

**Figure 9:**
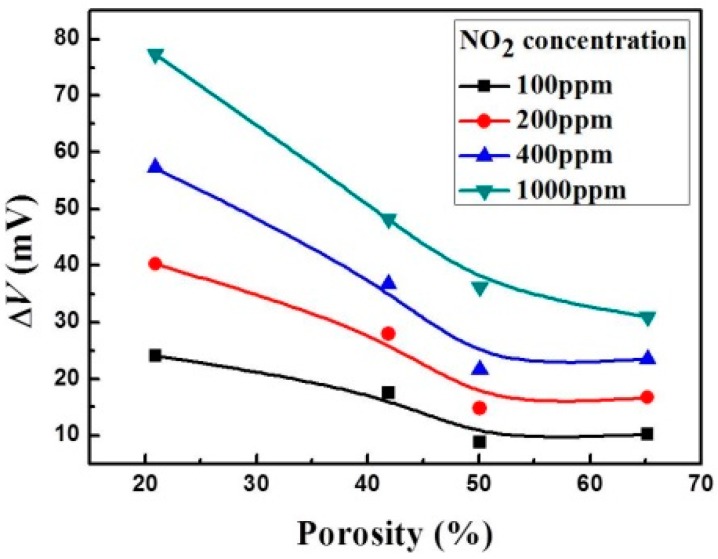
Dependence of potential difference on porosity of thick LSM95 layers.

The same thin LSM95 layers as SEs were co-fired with the four types of sensors with different amounts of carbon in the thick LSM95 REs (denoted as S-15LSM95, S-30LSM95, S-45LSM95 and S-60LSM95). Measurement were performed at 500 °C in various concentrations of NO_2_ (40–100 ppm). Figure 9 summarizes the Δ*V* at different RE porosities and NO_2_ concentrations, showing that the Δ*V* value decreased with increasing porosities. Therefore, low porosities of thick LSM95 as REs are preferred for better sensitivity of the new sensors. In particular, S-15LSM95 has the lowest porosity and exhibits the highest sensitivity.

### 3.4. Sensing Performance of Sensors with Different Sensing Materials

**Figure 10:**
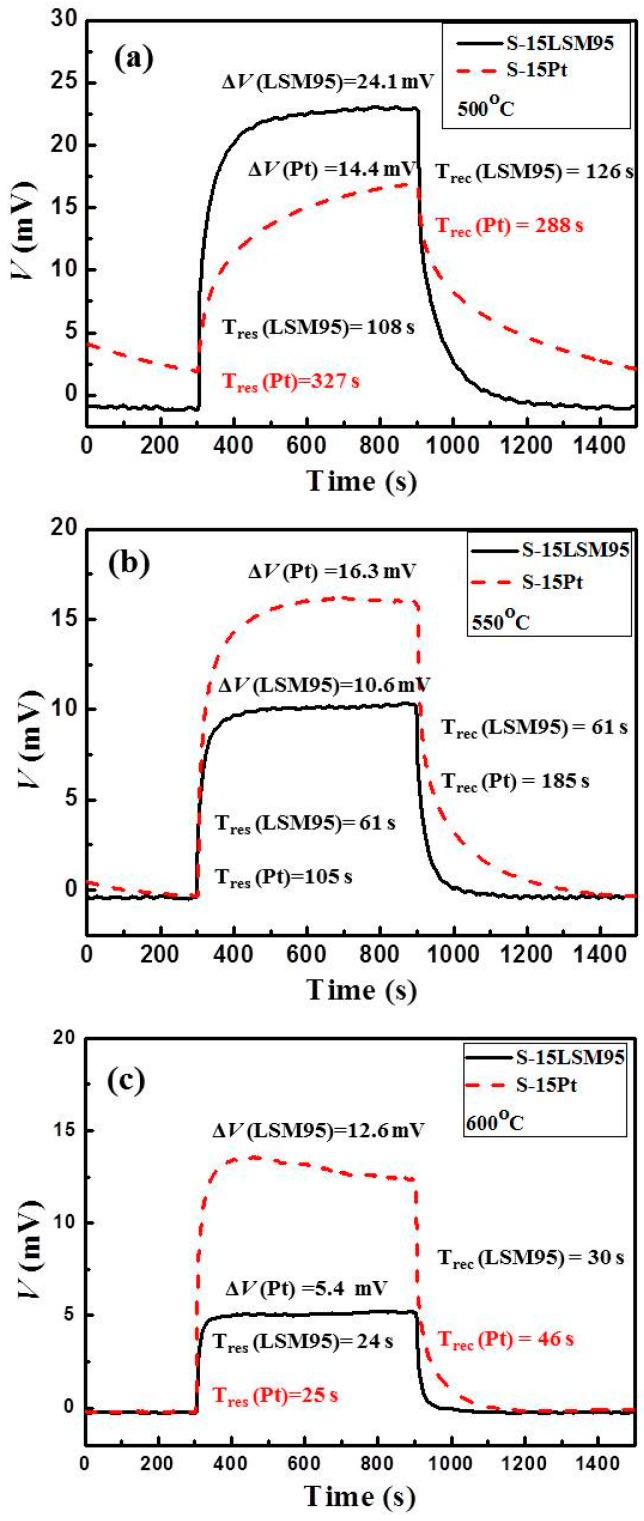
Response curves of S-LSM95 and S-Pt measured in the base gas and sample gas: (**a**) 500 °C, 100 ppm NO_2_; (**b**) 550 °C, 100 ppm NO_2_ and (**c**) 600 °C, 100 ppm NO_2_.

The thick LSM95 layers with 15 wt % carbon as pore former were used further investigations. Two sensors with the same thick LSM95 layer as REs and different sensing material as SEs were tested. The voltage (*V*) response transients to various concentrations of NO_2_ (40–1000 ppm) were recorded over the temperature range of 500–600 °C for S-15LSM95 and S-15Pt. Figure 10a–c present the transient curves of two sensors tested in base gas and sample gas (NO_2_ 100 ppm) at 500, 550 and 600 °C, respectively. Notably, we can observe that the response voltage for S-15LSM95 is higher than obtained for S-15Pt under the same measurement conditions (Figure 10a). Switching between the base gas and the sample gas, the *V* value of S-15LSM95 changes much more quickly than for S-15Pt, indicating faster response and recovery rates for S-15LSM95. In particular, the 90% response/recovery times are 108/126 s for S-15LSM95 and 327/288 s for S-15Pt, respectively. Increasing the measurement temperature to 550 and 600 °C yielded higher *V* values for S-Pt than for S-LSM95, with their transient curves compare in Figure 10b,c. The *V* value is close to zero under the base gas and increased more quickly than observed at 500 °C upon switching to the sample gas. The 90% response/recovery times at 550 °C are 61/61 s for S-15LSM95 and 105/185 s for S-15Pt, respectively.

With the increasing testing temperature, much more quick response rates are observed for the 90% response/recovery time at 600 °C, which are 24/25 s for S-15LSM95 and 30/46 s for S-15Pt, respectively. These results reveal that the S-15LSM95 always exhibits better response rates than the S-15Pt. 

**Figure 11:**
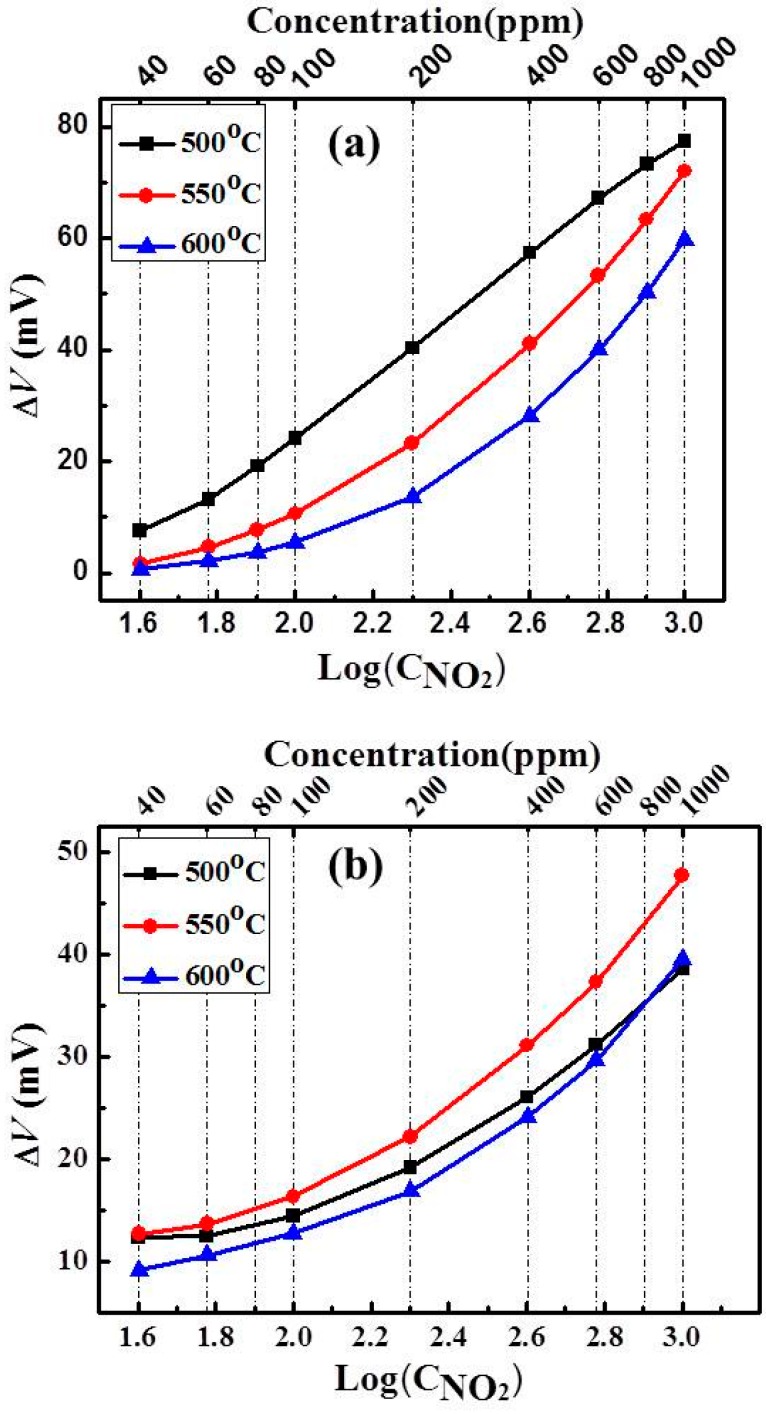
Dependence of response voltages on logarithm NO_2_ concentrations in the sample gas at 500–600 °C for the sensors: (**a**) S-LSM95 and (**b**) S-Pt.

Figure 11 summarizes the sensing characteristics of two sensors toward various NO_2_ concentrations from 40 to 1000 ppm in the sample gas at 500, 550 and 600 °C. It is seen that the measured voltage strongly depends on the SE material. The largest voltage values are 77 mV for S-15LSM95 and 48 mV for S-15Pt, both of which were obtained at 500 °C with the NO_2_ concentration is 1000 ppm in the sample gas. Comparison of the fitting results in Figure 6a,b indicates that S-LSM95 exhibited higher sensitivity, as evidenced by larger slopes, and much better linearity between the sensitivity and the logarithm of NO_2_ concentration. In particular, the highest linearity is 0.99 by fitting the results of S-15LSM95 at 500 °C with the largest sensitivity of 52 mV/decade.

**Figure 12:**
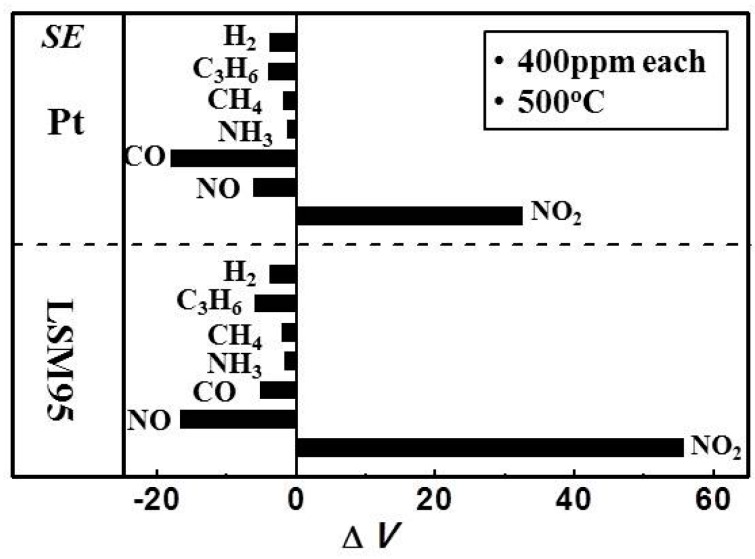
Cross-sensitivities of S-LSM95 and S-Pt to various gases (400 ppm) at 500 °C in the sample gas.

Note that various gases, other than NO_2_, including H_2_, C_3_H_6_, CH_4_, NH_3_, CO and NO exist in the car exhaust. Figure 12 compares the cross-sensitivities of the two sensors toward these gases at 400 ppm in sample gas, as measured at 500 °C. Both sensors exhibited the highest sensitivity to NO_2_. In contrast, the responses to most of the other gases are negligibly small (<6 mV) except for the values of 16–18 mV measured for S-Pt toward CO and S-15LSM95 toward NO, which are approximately half the value for the former and one third for the latter in NO_2_. Therefore, it can be concluded that S-15LSM95 exhibits better sensing characteristics than S-Pt at 500 °C.

### 3.5. Sensing Mechanism

**Figure 13:**
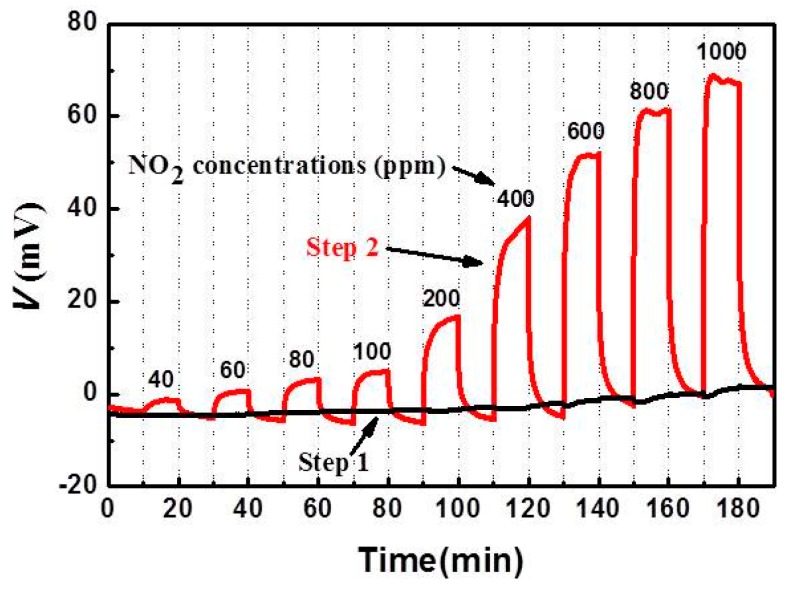
Sensing characteristics of SE and RE for S-LSM95 sensors measured at 500 °C in the two-chamber mode.

In order to better understand the sensing mechanism of these NO_2_ sensors, Figure 13 compares the response voltages measured in the dual-chamber mode as illustrated in Figure 2, where the NO_2_ concentration varied from 40 to 1000 ppm. As expected, the response voltages in step 1 and 2 are almost zero during 10 min with both thick and thin LSM95 layers exposed to the base gas. With thick LSM95 layers exposed to the sample gas (Step 1), the increase in the response voltage with increasing NO_2_ concentrations is negligible. In a vivid contrast, increasing NO_2_ concentrations yield a pronounced increase in the response voltage for NO_2_ sensors with the thin LSM95 layers exposed to the sample gas (Step 2). 

These results demonstrate that thin LSM95 layers are highly sensitive toward NO_2_ while thick LSM95 layers are almost catalytically inert. Therefore, the large response voltage values for S-15LSM95 in the single chamber mode should be ascribed to asymmetric configurations for the two porous LSM layers with thin layers active and thick layers inert.

It has been reported that traditional YSZ-based sensors commonly produce opposite signals toward NO_2_ and NO due to the opposite oxidation and reduction properties between them [27], as shown in electrochemical Equations (1) and (2) and gas phase reactions Equations (3) and (4):

(1) $${\text{NO}}_{2}+2\text{e}\to \text{NO}+{\text{O}}^{2-}$$

(2) $$\text{NO}+{\text{O}}^{2-}\to {\text{NO}}_{2}+2\text{e}$$

(3) $$2{\text{NO}}_{2}\to 2\text{NO}+{\text{O}}_{2}$$

(4) $$2\text{NO}+{\text{O}}_{2}\to 2{\text{NO}}_{2}$$

Recently, we have shown that the LSM95-supported NO sensors are based on a mixed-potential model [26]. Compared with previously reported YSZ-based NO_x_ sensors [6,28,29], the reference and sensing electrodes of S-15LSM95 are largely the same in terms of chemical composition and microstructure, but had very different thicknesses, which may play a critical role in determining the sensing performance. It is supposed that the gas phase reaction in Equation (3) proceeds continuously on the internal surfaces of porous LSM95 layers before reaching the triple-phase boundaries (TPBs) [30,31], where the electrochemical reaction of Equation (1) predominates and thereby produces the electrode potentials [32]. According to the experimental results, it can be found that thick LSM95 layers provided a very long diffusion pathway such that NO_2_ almost completely decomposed into NO along the TPBs, thereby producing quite low response *V* values. In other words, it is the electrochemical reaction occurring within the thin LSM95-SE that enables the desirable sensing performance due to the much shorter diffusion pathway. Therefore, the difference in the electrode thickness was the primary reason for the sensing signal towards NO_2_ between thin LSM95 layer and thick LSM95 layer since the NO_2_ and NO produced opposite signals in the electrochemical reactions on TPBs. Note that Miura [33] also reported that sensors produced smaller Δ*V* with thicker SEs. Similarly, the sensitivity of present sensors are decreased with increasing porosities of thick LSM95 layers (REs). Therefore, it is reasonable to conclude that the thick LSM95 layers minimize the adverse influence on the sensing characteristics due to NO_2_ within the REs.

## 4. Conclusions

New sensors consisted of thin 8YSZ electrolytes sandwiched between thick and thin LSM95 layers, simultaneously exposing to the same atmosphere, exhibited good sensing performance towards NO_2−_. The primary reason for such good sensing signals is the large difference in the diffusion pathways between the two electrodes, resulting from the very different thicknesses. Reducing the porosity for thick LSM95 REs help to increase the sensitivity. Compared with the Pt SEs, thin LSM95 SEs exhibit higher sensitivity, better linearity and faster response/recovery rates. Negligible responses of thick LSM95 layers toward NO_2_ demonstrate their great promise as an alternative to the Pt reference electrode in practical applications.

## References

[1] Mothe G., Castro M., Sthel M., Lima G., Brasil L., Campos L., Rocha A., Vargas H. (2010). Detection of Greenhouse Gas Precursors from Diesel Engines Using Electrochemical and Photoacoustic Sensors. Sensors.

[2] Qiu Q., Wu J., Liang G., Liu J., Chu G., Zhou G., Zhang D. (2015). Effects of simulated acid rain on soil and soil solution chemistry in a monsoon evergreen broad-leaved forest in southern China. Environ. Monit. Assess..

[3] Kampa M., Castanas E. (2008). Human health effects of air pollution. Environ. Pollut..

[4] Moos R. (2010). Catalysts as Sensors—A Promising Novel Approach in Automotive Exhaust Gas Aftertreatment. Sensors.

[5] Zheng Y., Li X., Dutta P.K. (2012). Exploitation of Unique Properties of Zeolites in the Development of Gas Sensors. Sensors.

[6] Miura N., Sato T., Anggraini S.A., Ikeda H., Zhuiykov S. (2014). A review of mixed-potential type zirconia-based gas sensors. Ionics.

[7] Moos R., Sahner K., Fleischer M., Guth U., Barsan N., Weimar U. (2009). Solid State Gas Sensor Research in Germany—A Status Report. Sensors.

[8] Lu G., Miura N., Yamazoe N. (1998). High-Temperature NO or NO_2_ Sensor Using Stabilized Zirconia and Tungsten Oxide Electrode. Ionics.

[9] Miura N., Akisada K., Wang J., Zhuiykov S., Ono T. (2004). Mixed-potential-type NOx sensor based on YSZ and zinc oxide sensing electrode. Ionics.

[10] Miura N., Shiraishi T., Shimanoe K., Yamazoe N. (2000). Mixed-potential-type propylene sensor based on stabilized zirconia and oxide electrode. Electrochem. Commun..

[11] Yang J.-C., Dutta P.K. (2007). Promoting selectivity and sensitivity for a high temperature YSZ-based electrochemical total NOx sensor by using a Pt-loaded zeolite Y filter. Sens. Actuators B Chem..

[12] Yang J.-C., Dutta P.K. (2007). High temperature amperometric total NOx sensors with platinum-loaded zeolite Y electrodes. Sens. Actuators B Chem..

[13] Yang J.-C., Dutta P.K. (2010). High temperature potentiometric NO_2_ sensor with asymmetric sensing and reference Pt electrodes. Sens. Actuators B Chem..

[14] Buciuman F.C., Patcas F., Menezo J.C., Barbier J., Hahn T., Lintz H.G. (2002). Catalytic properties of La_0.8_A_0.2_MnO_3_ (A = Sr, Ba, K, Cs) and LaMn_0.8_B_0.2_O_3_ (B = Ni, Zn, Cu) perovskites 1. Oxidation of hydrogen and propene. Appl. Catal. B Environ..

[15] Belardi R.M., Deseure J., Brant M.C., Matencio T., Domingues R.Z. (2009). Electrical study of cathodic activation and relaxation of La_0.8_Sr_0.2_MnO_3_. Ionics.

[16] Bredikhin S., Maeda K., Awano M. (2001). Electrochemical Cell with Two Layers Cathode for NO Decomposition. Ionics.

[17] Reinhardt G., Wiemhofer H.D., Gopel W. (1995). Electrode Reactions of La_0.8_Sr_0.2_MnO_3±δ_-Electrodes on Stabilized Zirconia with Oxygen and the Nitrogen Oxides NO and NO_2_. Ionics.

[18] Hwang H.J., Moon J.W., Awano M. (2003). Microstructure and NO decomposition behavior of sol-gel derived (La_0.8_Sr_0.2_)_0.95_MnO_3_/yttria-stabilized zirconia nanocomposite thin film. Mater. Res. Bull..

[19] Tietz F., Papadelis C., Tsiplakides D., Katsaounis A., Vayenas C.G. (2001). Temperature Programmed Oxygen Desorption of the Perovskites Series Ln_0.65_Sr_0.3_Mn_0.8_Co_0.2_O_3_ (Ln = La-Gd). Ionics.

[20] Charpentier P., Fragnaud P., Schleich D.M., Denos Y., Gehain E. (1998). Preparation of Thin Film SOFCs Working at Reduced Temperature. Ionics.

[21] Nagde K.R., Bhoga S.S. (2010). Study of mechanochemically prepared nanocrystalline La_0.8_Sr_0.2_MnO_3_. Ionics.

[22] Zhang S., Bi L., Zhang L., Yang C., Wang H., Liu W. (2009). Fabrication of cathode supported solid oxide fuel cell by multi-layer tape casting and co-firing method. Int. J. Hydrogen Energy.

[23] Zhao C., Liu R., Wang S., Wen T. (2009). Fabrication of a large area cathode-supported thin electrolyte film for solid oxide fuel cells via tape casting and co-sintering techniques. Electrochem. Commun..

[24] Yuan C., Liu Y., Zhou Y., Zhan Z., Wang S. (2013). Fabrication and characterization of a cathode-support solid oxide fuel cell by tape casting and lamination. Int. J. Hydrogen Energy.

[25] Liu Y., Wang S., Qian J., Xin X., Zhan Z., Wen T. (2013). A novel catalytic layer material for direct dry methane solid oxide fuel cell. Int. J. Hydrogen Energy.

[26] Zou J., Liu X., Jin H., Zhan Z., Jian J. (2015). NO_2_ sensing properties of electrode-supported sensor by tape casting and co-firing method. Ionics.

[27] Zhuiykov S., Nakano T., Kunimoto A., Yamazoe N., Miura N. (2001). Potentiometric NOx sensor based on stabilized zirconia and NiCr_2_O_4_ sensing electrode operating at high temperatures. Electrochem. Commun..

[28] Elumalai P., Miura N. (2005). Performances of planar NO_2_ sensor using stabilized zirconia and NiO sensing electrode at high temperature. Solid State Ion..

[29] Kading S., Jakobs S., Guth U. (2003). YSZ-cells for potentiometric nitric oxide sensors. Ionics.

[30] Zhu J.J., Thomas A. (2009). Perovskite-type mixed oxides as catalytic material for NO removal. Appl. Catal. B Environ..

[31] Huang T.J., Wang C.H. (2011). Effect of temperature and NOx concentration on nitric oxide removal from simulated lean-burn engine exhaust via electrochemical-catalytic cells. Chem. Eng. J..

[32] Xiong W., Kale G.M. (2007). Electrochemical NO_2_ sensor using a NiFe_1.9_Al_0.1_O_4_ oxide spinel electrode. Anal. Chem..

[33] Elumalai P., Plashnitsa V.V., Ueda T., Hasei M., Miura N. (2006). Dependence of NO_2_ sensitivity on thickness of oxide-sensing electrodes for mixed-potential-type sensor using stabilized zirconia. Ionics.

